# The Springtime Influence of Natural Tropical Pacific Variability on the Surface Climate of the Ross Ice Shelf, West Antarctica: Implications for Ice Shelf Thinning

**DOI:** 10.1038/s41598-018-30496-5

**Published:** 2018-08-10

**Authors:** Kyle R. Clem, Andrew Orr, James O. Pope

**Affiliations:** 1 Institute of Earth, Ocean, and Atmospheric Sciences, Rutgers, The State University of New Jersey, New Brunswick, New Jersey USA; 2 British Antarctic Survey, Cambridge, United Kingdom

## Abstract

Observational records starting in the 1950s show West Antarctica is amongst the most rapidly warming regions on the planet. Together with increased intrusions of warm circumpolar deep water (CDW) onto the continental shelf due to local wind forcing (the primary mechanism in recent decades), this has resulted in enhanced surface and basal melting of floating ice shelves and an associated acceleration and thinning of West Antarctic outlet glaciers, increasing the rate of global sea level rise. In this study, it is shown that during the austral spring season, significant surface warming across West Antarctica has shifted westward to the Ross Ice Shelf in recent decades in response to enhanced cyclonic circulation over the Ross Sea. These circulation changes are caused by a Rossby wave train forced by increasing sea surface temperatures in the western tropical Pacific, which is tied to the springtime shift of the Interdecadal Pacific Oscillation (IPO) to its negative phase after 1992. While the local wind trends enhance warm air advection and surface warming across the Ross Ice Shelf, the strong easterly component of the wind trends reduces the likelihood for intrusions of CDW onto the continental shelf in this region. This suggests that during spring there are competing mechanisms of surface and basal melting of the Ross Ice Shelf, both of which are closely tied to natural tropical Pacific decadal variability. Moreover, that the projected transition of the IPO back to its positive phase in the coming decade, though likely to reduce surface warming on the Ross Ice Shelf, could increase the risk of disintegration of Ross Sea ice shelves due to increased intrusions of CDW and enhanced basal melting.

## Introduction

Observational records beginning in the late 1950s recorded an increase in surface air temperature (SAT) over West Antarctica that was more than twice as fast as the global average^1–3^. The warming is strongest and most widespread during the austral winter and spring seasons^4–11^. In addition to the warming, there has been thinning of ice shelves in the Amundsen Sea Embayment (ASE) and an associated acceleration and thinning of West Antarctic outlet glaciers which has resulted in a negative trend in ice-sheet mass balance and a faster rate of global sea level rise^12–18^. This thinning is predominately due to increased influxes of warm circumpolar deep water (CDW) along glacial troughs caused by increases in local westerly winds and upwelling over the continental shelf break^15,19–23^, as well as increasing SAT which causes surface melting that can lead to meltwater ponding, hydrofracturing, and ice shelf collapse^24–27^; surface melting has been the leading contributor to the disintegration of ice shelves in the Antarctic Peninsula^28–30^, and is likely to increase dramatically in importance in the ASE region by the end of the century^31,32^.

**Figure 1:**
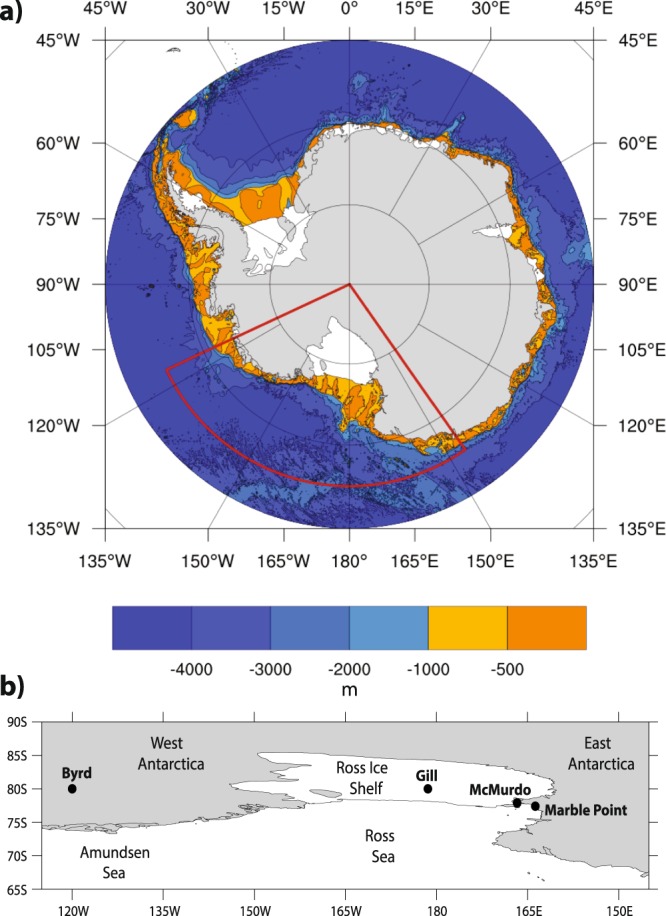
Map of the study area showing (**a**) bathymetry from Bedmap2^87^ with glacial troughs (~1000 meters) displayed in light orange and (**b**) locations of the weather stations used.

However, a closer examination reveals that SAT trends across West Antarctica exhibit considerable decadal variability and, despite their strong magnitude, lie within the bounds of natural variability^33,34^. Multiple gridded temperature datasets suggest that spring warming has continued and even intensified across West Antarctica in recent decades, but it has shifted westward to the Ross Ice Shelf^11,35,36^. While no major surface melting has been documented in this region during spring, surface melting is commonly observed over the Ross Ice Shelf during summer^24–27^, and it is likely to become more frequent by the end of the century^31,32^. This westward shift in spring warming is confirmed by the sparse station observations in the region, which show over recent decades a reduction in warming at Byrd station in central West Antarctica^3^, while McMurdo station located on the western Ross Ice Shelf exhibits a large springtime warming trend that is stronger than any Antarctic station during any season^37^. The cause of the shift in spring warming is suggested to be tied to anomalous meridional winds^3^ due to an increase in cyclonic circulation offshore over the Ross Sea^35,38,39^, resulting in local changes to thermal advection^35,36^ and sea ice concentration^10^. However, anomalous easterlies over the continental shelf break resulting from the strengthened cyclonic circulation would be expected to decrease intrusions of CDW onto the inner continental shelf and thus reduce basal melting of ice shelves in the Ross Sea region^20,40^, i.e. there are competing impacts on the stability of the ice shelves in this region as an increase in surface melting (and related ponding and hydrofracturing) due to warming may be offset by a reduction in basal melting. See Fig. 1 for a map showing the station locations, as well as the continental shelf break.

Previous modeling^10,41–44^ and observational^35,36^ studies have suggested that during spring the increased cyclonic circulation is linked to tropical variability, namely the transition of the Interdecadal Pacific Oscillation (IPO)^45–47^ (similar to the Pacific Decadal Oscillation) to its negative phase after the 1990s^35,43^ as well as increasing sea surface temperature (SST) in the tropical North Atlantic^48^. The IPO is a naturally occurring mode of decadal variability in the El Niño-Southern Oscillation (ENSO)^49–51^, meaning that these changes may be part of natural variability^52^. Despite these findings, the precise mechanism by which the negative IPO phase causes an increase in cyclonic circulation over the Ross Sea remains controversial; previous studies suggest a variety of mechanisms are responsible, including negative SST anomalies over the eastern tropical Pacific^43^, increased deep convection in the South Pacific Convergence Zone^36^, increased wave activity from the central tropical Pacific^35^, and a wave train emanating from the tropical Atlantic^48^. Our present work addresses this important gap by analyzing gridded datasets and station observations in conjunction with atmosphere-only climate model simulations to show a robust mechanism linking western tropical Pacific SST variability associated with the IPO to the recently observed spring circulation and temperature changes across the Ross Sea and Ross Ice Shelf, as well as local zonal winds over the continental shelf break, and by extension potential atmospheric drivers of ice shelf thinning.

### Ross Ice Shelf Spring Temperature and Circulation Linked to Tropical Pacific Variability

**Figure 2:**
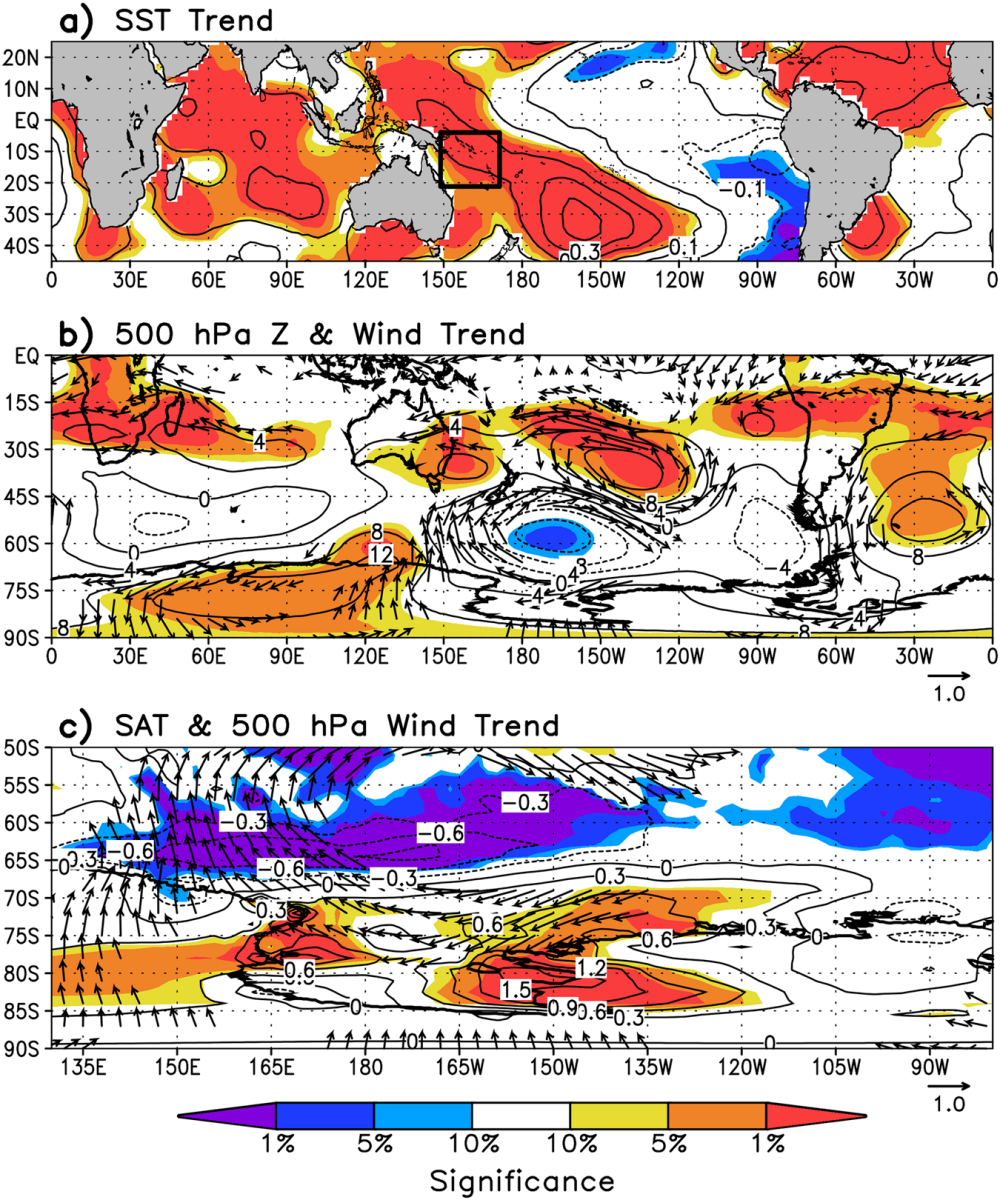
September-October-November (SON) linear trend between 1979 and 2014 of (**a**) NOAA’s ERSSTv4 tropical SST, (**b**) Southern Hemisphere ERA-Interim 500 hPa geopotential height and wind, and (**c**) western West Antarctic ERA-Interim 2 m temperature and 500 hPa wind. Shading indicates where trends are significant at *p* < 0.10, *p* < 0.05, and *p* < 0.01 as indicated by color bar at the bottom. Wind vectors are in units of ms^−1^ decade^−1^ and are drawn only if at least one component is significant at *p* < 0.10. Contour interval is 0.1 °C decade^−1^ in (**a**), 4 m decade^−1^ in (**b**), and 0.3 °C decade^−1^ in (**c**).

**Figure 3:**
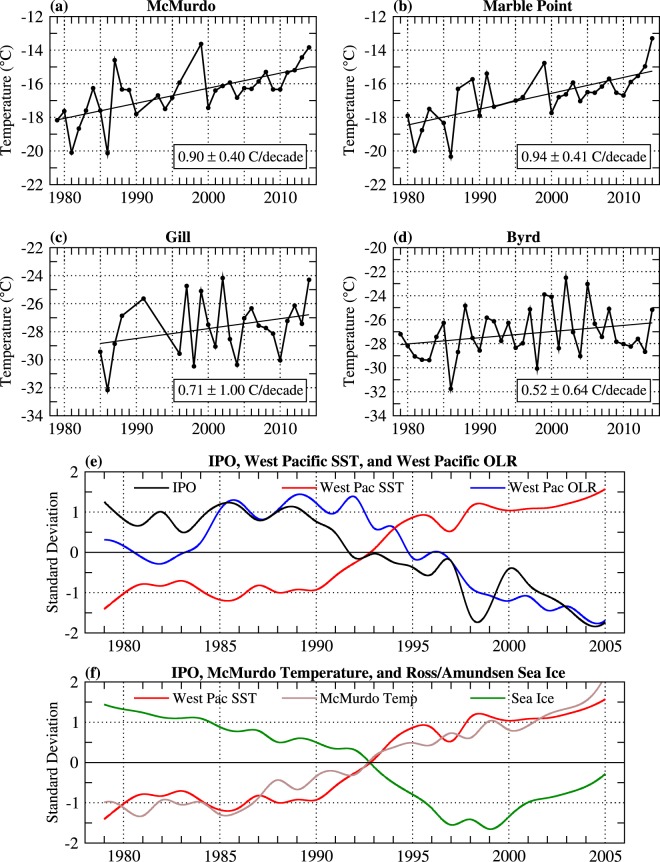
Top four panels are the time series of observed SON temperatures from weather stations located on the Ross Ice Shelf and central West Antarctica between 1979 and 2014. (**a**) McMurdo, (**b**) Marble Point, (**c**) Gill, and (**d**) Byrd^3^. Bottom two panels are standardized SON 10-year running averages of (**e**) IPO index^47^ and western tropical Pacific SST and outgoing longwave radiation (OLR) averaged over 150–170°E, 5–20°S, and (**f**) western tropical Pacific SST, ERA-Interim 2 m temperature grid cell located nearest to McMurdo (78°S, 166.5°E), and sea ice concentration in the southeast Ross Sea/southwest Amundsen Sea averaged over 164.5–130.5°W, 68.5–73.5°S (see Fig. S1 for sea ice region).

During the post-1979 modern satellite era, spring tropical SSTs increased by up to 0.3 °C decade^−1^ over the western tropical Pacific as well as the tropical Indian and North Atlantic basins, while the eastern tropical Pacific cooled 0.1–0.2 °C decade^−1^ (Fig. 2a). Contemporaneously, reanalysis trends show a marked strengthening in cyclonic activity over the Ross Sea resulting in strengthened easterly winds over the continental shelf break, which is tied to a decrease in cyclone central pressures^38^ and 500 hPa geopotential height in this region (Fig. 2b). Additionally, SAT increased across western West Antarctica and the western and eastern portions of the Ross Ice Shelf by 0.5–1.5 °C decade^−1^ (aligning with increased northeasterly winds and reductions in sea ice concentration (Fig. S1)), but remained unchanged across central and eastern West Antarctica (Fig. 2c). The reanalysis trends are broadly in agreement with station temperature trends (Fig. 3), with warming of approximately 0.9 °C decade^−1^ (significant at *p* < 0.01) observed at McMurdo and Marble Point (located on the western Ross Ice Shelf), while weaker and statistically insignificant warming is measured at Byrd and Gill (located in central West Antarctica and the central Ross Ice Shelf).

**Figure 4:**
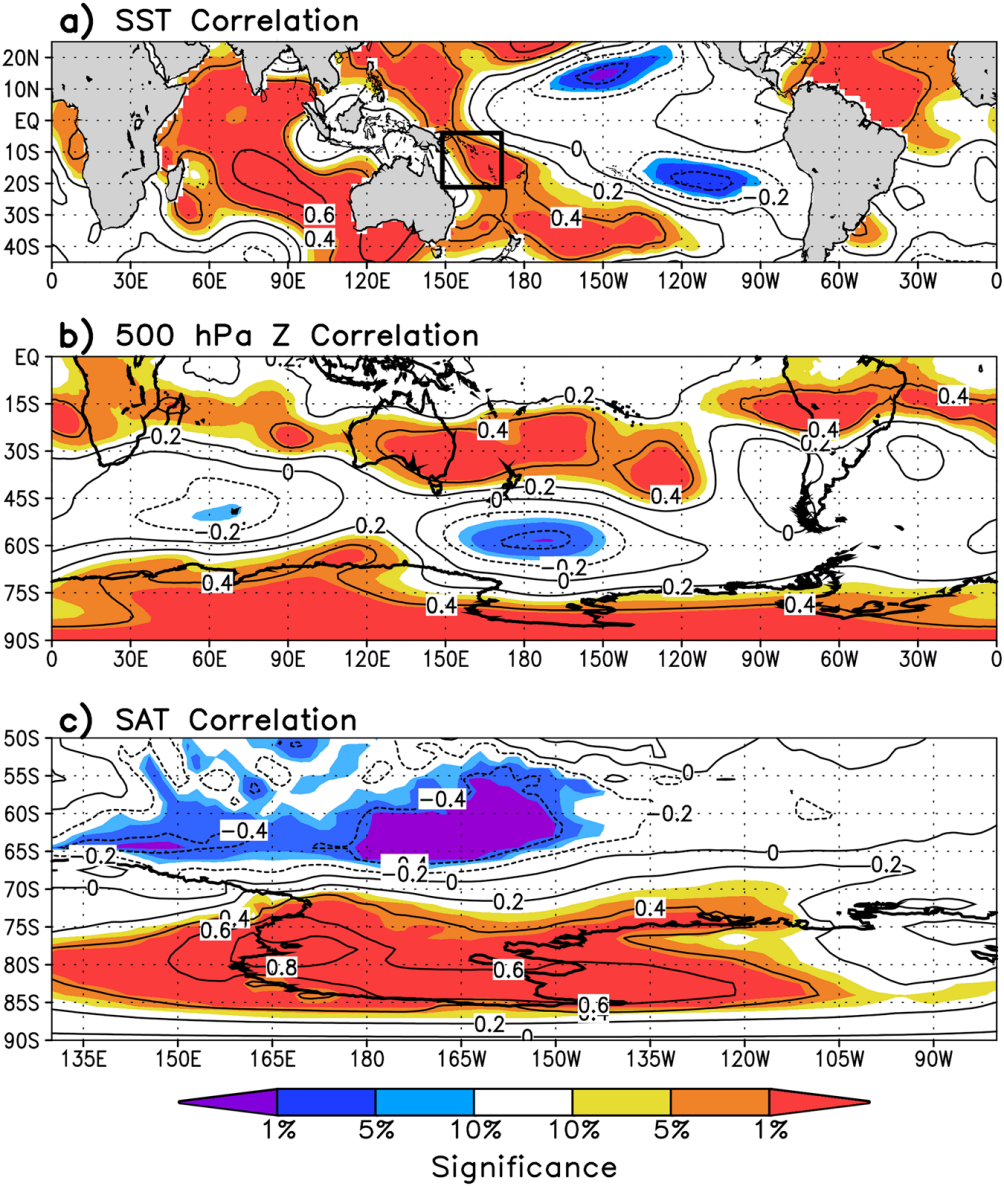
SON correlations over 1979–2014 of McMurdo temperature with (**a**) NOAA’s ERSSTv4 tropical SST, (**b**) Southern Hemisphere ERA-Interim 500 hPa geopotential height, and (**c**) western West Antarctic ERA-Interim 2 m temperature. Shading indicates where correlations are statistically significant and is drawn as in Fig. 2. Contour interval is 0.2.

A difficulty in using the station measurements in this region is the incomplete temperature records, many of which have gaps^3^. We therefore use the reanalysis 2 m temperature at the grid cell nearest to McMurdo station to form a continuous temperature record at McMurdo for further analysis (termed McMurdo temperature hereafter). This site is chosen because McMurdo has the most complete record in the region, which is used to constrain the reanalysis, and it also shows strong autocorrelation with reanalysis 2 m temperatures throughout the Ross Ice Shelf and western West Antarctica (see Fig. 4c), i.e. it is a reliable “complete” temperature record representative of the Ross Ice Shelf. The autocorrelation with SAT arises due to the predominant local circulation in this region termed the Ross Ice Shelf airstream^53^, which through an increase in katabatic winds and barrier winds results in uniform SAT anomalies across the Ross Ice Shelf^54–58^. The Ross Ice Shelf airstream occurs with cyclonic circulation in the Ross Sea, which strengthens easterly winds across much of the Ross Ice Shelf which locally enhances katabatic winds and barriers winds^59–62^, as evident in Fig. 2c.

The tropical Pacific SST trends shown in Fig. 2a resemble a La Niña pattern^35^ and the negative phase of the IPO^63^, implying the post-1979 trends are closely tied to the shift in the IPO to its negative phase around 1992 during spring (Fig. 3e). Closely following this transition is an increase in SST and deep convection (evidenced by a decrease in outgoing longwave radiation) over the western tropical Pacific (Fig. 3e; averaged over the box shown in Fig. 2a, between 150–170°E and 5–20°S). Note that the correlation between our west Pacific SST box and the IPO is −0.79. McMurdo temperature shows a nearly identical transition from cold anomalies to warm anomalies around 1992, and similarly nearby sea ice concentrations over the southern Ross Sea and Amundsen Sea (Fig. 3f; averaged over the box shown in Fig. S1) transition from positive to negative anomalies around 1992. McMurdo temperature shows strong covariability with western tropical Pacific SST on decadal timescales, as demonstrated by the time series (Fig. 3f), as well as on interannual timescales (e.g. Fig. 4a). McMurdo temperature is significantly correlated with negative geopotential heights over the northern Ross Sea, as well as positive geopotential heights in the subtropical western Pacific and across the interior continent (Fig. 4b). It is noteworthy that McMurdo temperature is positively correlated with SSTs in the southwest Indian Ocean (Fig. 4a), but closer examination reveals that during spring this region is characterized by large-scale subsidence (Fig. S2) and therefore unable to generate the necessary deep convection to force a Rossby wave into high southern latitudes^64^. Altogether, the correlations imply a close linear relationship between Ross Ice Shelf SAT (and by extension local circulation), enhanced cyclonic circulation in the Ross Sea, and western tropical Pacific SSTs that are strongly influenced by the IPO.

### Model Response to Anomalous Western Tropical Pacific Warming in Spring

To test for a causal relationship linking the observed western tropical Pacific warming to the cyclonic circulation in the Ross Sea, a control experiment was performed by running the HadGEM3A atmosphere-only climate model^65^ for 30 years (following a 10-year spin-up) forced by pre-industrial SSTs. We then performed a perturbation experiment by repeating the control run, but with the addition of a 2 °C SST anomaly in the western tropical Pacific (150–170°E, 5–20°S; box in Figs 2a and 4a). In order to emphasize the impact of the SST anomaly, the influence of natural variability in the simulations was reduced by driving the control run with a repeating mean annual cycle of climatologically averaged SSTs, i.e. damping the effects such as e.g. ENSO.

**Figure 5:**
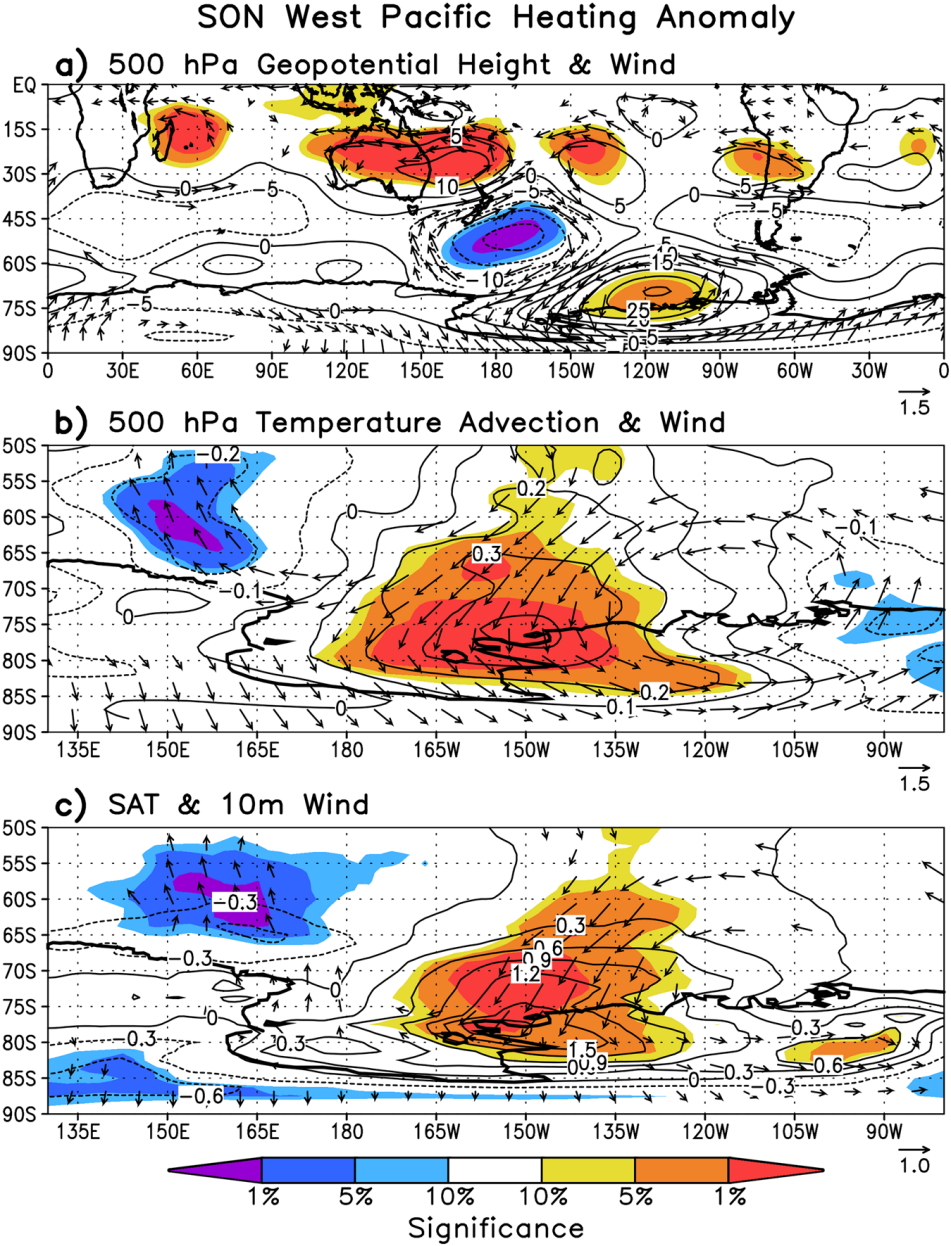
SON difference in HadGEM3A 30-year average simulation forced by a +2 °C western tropical Pacific SST heating anomaly minus HadGEM3A 30-year average climatology run. (**a**) 500 hPa geopotential height and 500 hPa wind, (**b**) 500 hPa meridional temperature advection $-v\frac{\partial T}{\partial y}$ and wind, and (**c**) 2 m temperature and 10 m wind. Shading indicates where differences are statistically significant and is drawn as in Figs 2 and 4. Wind vectors, in ms^−1^, are shown only if at least one component is significant at *p* < 0.10. Contour interval for (**a**) 5 m, (**b**) 0.1 °C day^−1^, and (**c**) 0.3 °C.

The climatological difference in 500 hPa geopotential height between the perturbed and control experiments during spring (Fig. 5a) is broadly consistent with the circulation pattern evident in the reanalysis trends (Fig. 2b), depicting an anomalous wave train emanating from the western tropical Pacific region, which propagates southeastward into the Ross and Amundsen Seas. The perturbation experiment reproduces the springtime intensification of cyclonic circulation over the Ross Sea seen in the reanalysis trends (Fig. 2b), as well as the associated strengthening of northeasterly winds over the southeast Ross Sea and the eastern Ross Ice Shelf. The northeasterly winds originate at middle latitudes over the Southern Ocean (near 50°S) providing favorable conditions for transporting relatively warm maritime air poleward toward the continent, which is evidenced by the warm air advection (Fig. 5b) and positive SAT anomalies (Fig. 5c) aligning with the northeasterly winds. Along the western Ross Ice Shelf near McMurdo there are anomalous southerly winds at the surface (Fig. 5c), indicative of strengthened barrier winds associated with the Ross Ice Shelf airstream (which is common with cyclonic circulation in the Ross Sea). While the winds largely favor warming across the Ross Ice Shelf, the anomalous easterlies over the continental shelf break would be expected to reduce upwelling and intrusions of CDW extending from the western ASE to the Ross Ice Shelf.

The model difference also shows the tropically-forced wave train is associated with an anti-cyclonic circulation in the Amundsen Sea, which is not apparent in the reanalysis trends (Fig. 2b). This is likely due to the damping of natural variability/ENSO in the experiments, consistent with ref.^35^ which showed springtime La Niña forcing causes anomalous cyclonic circulation in the Amundsen Sea, which cancels the IPO-forced anti-cyclonic circulation (as seen in the model). To test this, we re-calculated the spring trends after removing years when La Niña occurred in isolation without a negative IPO event (2010, 1988, 2000, 1998) in order to isolate the negative IPO forcing without La Niña. The new reanalysis trends (Fig. S3) depicting IPO forcing are in much better agreement with the model simulation. In particular, the new reanalysis trends show a more intense cyclonic circulation in the Ross Sea, an anti-cyclonic circulation in the Amundsen Sea, 0.2–0.4 °C decade^−1^ stronger warming over the eastern Ross Ice Shelf and 0.1–0.2 °C decade^−1^ stronger warming near McMurdo, and strengthened easterlies over the continental shelf break. This provides additional evidence that the circulation seen in the simulation is consistent with variability in the IPO and not La Niña, and tropical Pacific decadal variability (not ENSO) is the primary driver of the recent spring changes in circulation, SAT, and local wind forcing of CDW across the Ross Ice Shelf.

## Discussion and Conclusions

This study demonstrates strong covariability of spring circulation and temperature over the Ross Ice Shelf with remote forcing from the western tropical Pacific tied to natural variability in the springtime IPO. Performing a sensitivity experiment with a climate model shows that positive SST anomalies in the western tropical Pacific results in a Rossby wave train that reproduces the observed increase in cyclonic circulation in the Ross Sea and the associated westward shift of West Antarctic spring warming to the Ross Ice Shelf. While the circulation causes surface warming on the Ross Ice Shelf, the anomalous easterlies over the continental shelf break would likely be associated with a reduction in CDW intrusions and basal melt in this region. Therefore, the post-1979 springtime circulation and temperature trends across the Ross Ice Shelf are primarily a result of the post-1992 transition of the IPO to its negative phase, and future springtime changes, including both surface melt (via increases in SAT) and potentially basal melt (via intrusions of CDW caused by local zonal wind forcing), will be modulated by natural tropical Pacific SST variability, though more work is needed to quantify its influence on ice shelf stability.

While the warming over the eastern Ross Ice Shelf is caused by strengthened northeasterly winds and warm air advection (consistent with ref.^35^), which is also likely tied to the springtime reduction in sea ice concentration in the southeast Ross Sea and southwest Amundsen Sea (Fig. S1a), warming over the western Ross Ice Shelf near McMurdo is more tied to localized winds associated with the Ross Ice Shelf airstream^66,67^. This is demonstrated further in Fig. S4 showing positive temperature anomalies at McMurdo and Marble Point are strongly correlated with easterly wind anomalies over the northern Ross Ice Shelf, as seen in the model. Although the model experiment reproduces the overall synoptic circulation pattern in the region, it does not explicitly capture the surface warming along the western Ross Ice Shelf, likely because its coarse resolution (around 150 km) is insufficient to resolve the localized barrier winds, as well as possible issues with its representation of the boundary layer^68^.

It should also be noted that the cyclonic circulation in the Ross Sea produces anomalous southerly wind and cold air advection in the western Ross Sea where cooling and sea ice expansion has been observed in spring during the 21st century^33,43,69,70^. Therefore, a portion of the recent springtime sea ice expansion in the Ross Sea is likely tied to warming of the western tropical Pacific associated with the IPO. Moreover, this circulation pattern is markedly different than that shown in ref.^43^ during 2000–2014; ref.^43^ linked recent sea ice expansion in the Ross Sea to enhanced cyclonic circulation farther east over the Amundsen and Bellingshausen Seas. Therefore IPO-related SST variability generates two distinct wave trains during spring, one from the western tropical Pacific and one from the eastern tropical Pacific^43^, each of which affects different aspects of West Antarctic climate and sea ice. Also induced by the western tropical Pacific wave train is anti-cyclonic circulation in the Amundsen Sea (which is partially offset in the real world by an increase in La Niña forcing during periods of negative IPO^44^) that produces cold air advection over continental West Antarctica east of 105°W (Fig. 5b), which helps explain the reduction in surface warming observed there after the 1990s^71,72^.

Lastly, recent work by ref.^24^ showed El Niño causes enhanced surface melting on the Ross Ice Shelf during summer, while here we show negative phases of the IPO may also lead to enhanced melting but during the spring season, especially as temperatures in the polar regions continue to increase at a greater rate than the global average (i.e. polar amplification) and surface melt events on the Ross Ice Shelf become more common^31^. The results presented here along with ref.^24^ indicate that multiple aspects of natural tropical Pacific variability play important roles in modulating surface melting of the Ross Ice Shelf and potentially influxes of CDW onto the continental shelf due to local zonal wind forcing. In the coming decades, as the IPO likely shifts to its positive phase^73^, an increase in anti-cyclonic circulation over the Ross Sea is expected during spring, which would reduce the surface warming but increase local westerly wind anomalies over the continental shelf. Such changes in surface melt and ocean-driven basal melt therefore oppose each other in this region, and the relative importance of these competing mechanisms for future Ross Ice Shelf stability will require further cross-disciplinary study involving glaciologists and oceanographers to fully understand the influence of the IPO on the stability of the Ross Ice Shelf (which is outside the scope of this investigation). In particular, more work is needed to quantify the magnitude of basal melt one would expect from shifts in the IPO, and if a reduction in surface warming would offset any of the potential ice shelf thinning. Similar cross-disciplinary work has been proven successful in the ASE^23,40^, though similarly more work is needed to fully quantify the magnitude of basal melt resulting from remote tropical forcing of local zonal winds in these regions^74^. To fully understand this threat will also require improving the projection of natural modes of decadal tropical variability and their associated teleconnections over the South Pacific.

## Methods

Atmospheric circulation and SAT data are from the European Centre for Medium-Range Weather Forecasts (ECMWF) Interim reanalysis^75^ (ERA-Interim). Other reanalyses yield similar results during spring (as discussed by ref.^35^), but ERA-Interim has been shown to be most reliable over high southern latitudes^76–78^. Sea surface temperature data are from the National Oceanic and Atmospheric Administration (NOAA) Extended Reconstructed SST version 4 (ERSSTv4) dataset^79–81^, sea ice concentration data are from the Hadley Centre Sea Ice and SST dataset^82^, and outgoing longwave radiation data are from the NOAA Interpolated Outgoing Longwave Radiation dataset^83^. Antarctic weather station temperature data are from the Antarctic READER project^84^. We monitor variability in the IPO using the tripole index^47^.

Linear trends were calculated using a linear least squares regression analysis and relationships were investigated using linear Pearson’s correlation analysis. Statistical significance of trends, correlations, and differences in the model climatologies were calculated using a two-tailed Student’s *t* test.

The atmosphere-only third generation Hadley Centre Global Environmental Model^85^ (HadGEM3A) utilizes the Global Atmosphere 4.0 and Global Land 4.0 configurations of physics and dynamics^65^. The model experiments were run at N96 resolution (equivalent to 1.875° latitude × 1.25° longitude), with 85 vertical levels up to a height of 85 km, partitioned with 50 levels below 18 km and 35 levels between 18 km and 85 km^65^. Both experiments were forced by pre-industrial concentrations of greenhouse gases, representative of the 1860s. The SST and sea ice concentration fields for both experiments are prescribed and also representative of pre-industrial conditions, which are generated from the HadGEM2-CC coupled climate model historical simulation submitted to phase 5 of the Coupled Model Intercomparison Project^86^. Both experiments were run for 30 years in response to a repeating annual cycle of climatologically averaged SSTs and sea ice to remove sources of natural variability, such as ENSO.

## Electronic supplementary material

Supporting information
