# Supplementary material for: The Springtime Influence of Natural Tropical Pacific Variability on the Surface Climate of the Ross Ice Shelf, West Antarctica: Implications for Ice Shelf Thinning
Source: Sci Rep. 2018 Aug 10;8:11983. doi: 10.1038/s41598-018-30496-5 (PMC6086883; doi:10.1038/s41598-018-30496-5)
Supplement: Supplementary file 1 — Supporting information [file 41598_2018_30496_MOESM1_ESM.pdf]

Supporting information for:

The Springtime Influence of Natural Tropical Pacific Variability on the Surface Climate of the Ross Ice Shelf, West Antarctica: Implications for Ice Shelf Thinning

Kyle R. Clem[1*], Andrew Orr[2], and James O. Pope[2]

[1*] Institute of Earth, Ocean, and Atmospheric Sciences, Rutgers, The State University of New Jersey, New Brunswick, New Jersey.

[2] British Antarctic Survey, Cambridge, United Kingdom.

Contents of this file:

Figures S1 to S4

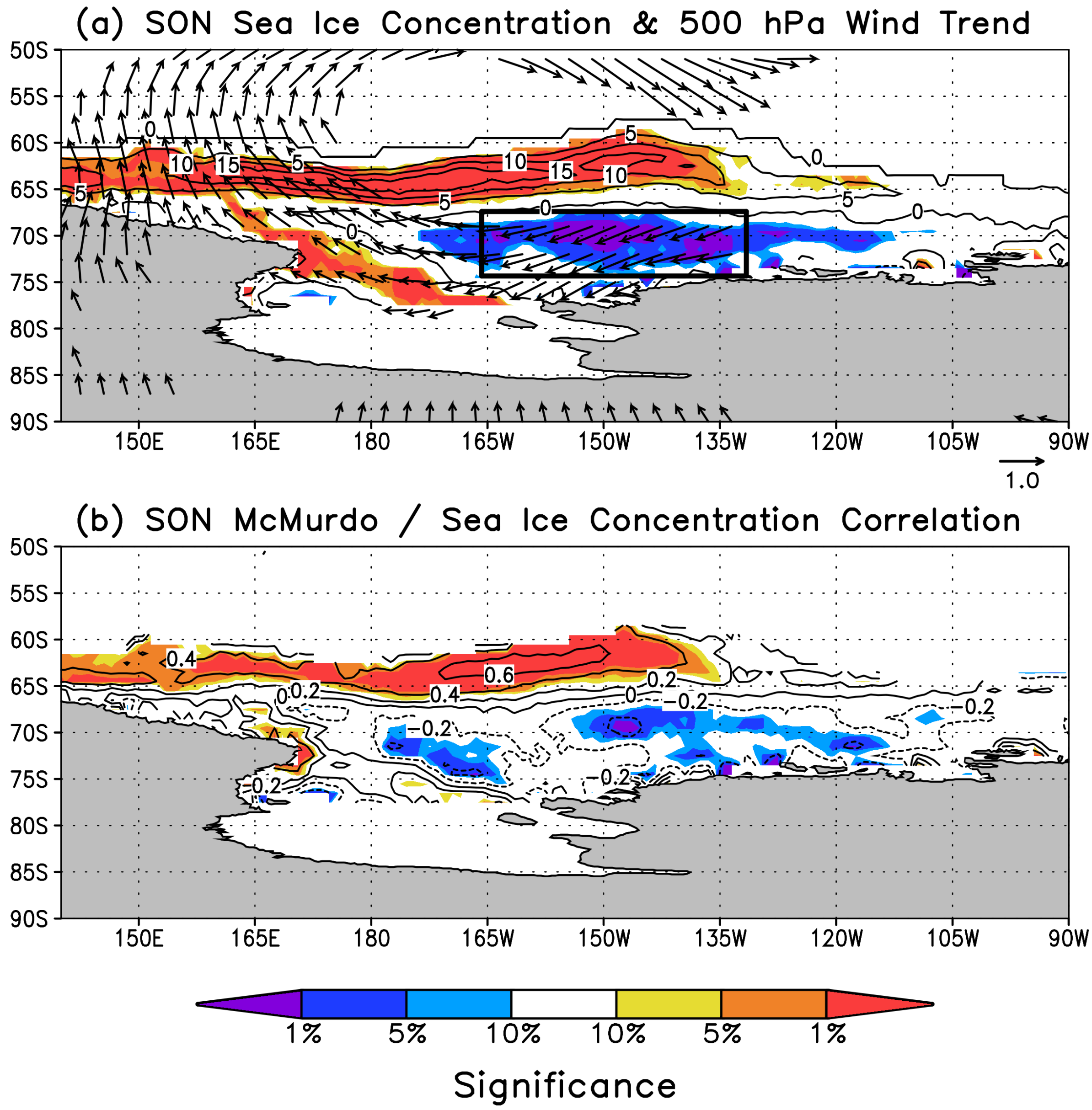

Supplementary Figure 1. Top panel (a) are September-October-November (SON) linear trends over 1979-2014 of western West Antarctic HadISST sea ice concentrations and 500 hPa winds. Shading in (a) indicates where trends are significant at p<0.10, p<0.05, and p<0.01 as indicated by color bar at the bottom. Vectors are shown only if at least one component is significant at p<0.01. Black box in (a) denotes the region used for the time series in Fig. 3f. Contour interval in (a) is 5% decade^{-1}. Bottom panel (b) is the SON correlation over 1979-2014 of the ERA-Interim McMurdo temperature with HadISST sea ice concentrations. Shading in (b) indicates where correlations are significant at p<0.10, p<0.05, and p<0.01 as indicated by color bar at the bottom. Contour interval in (b) is 0.2.

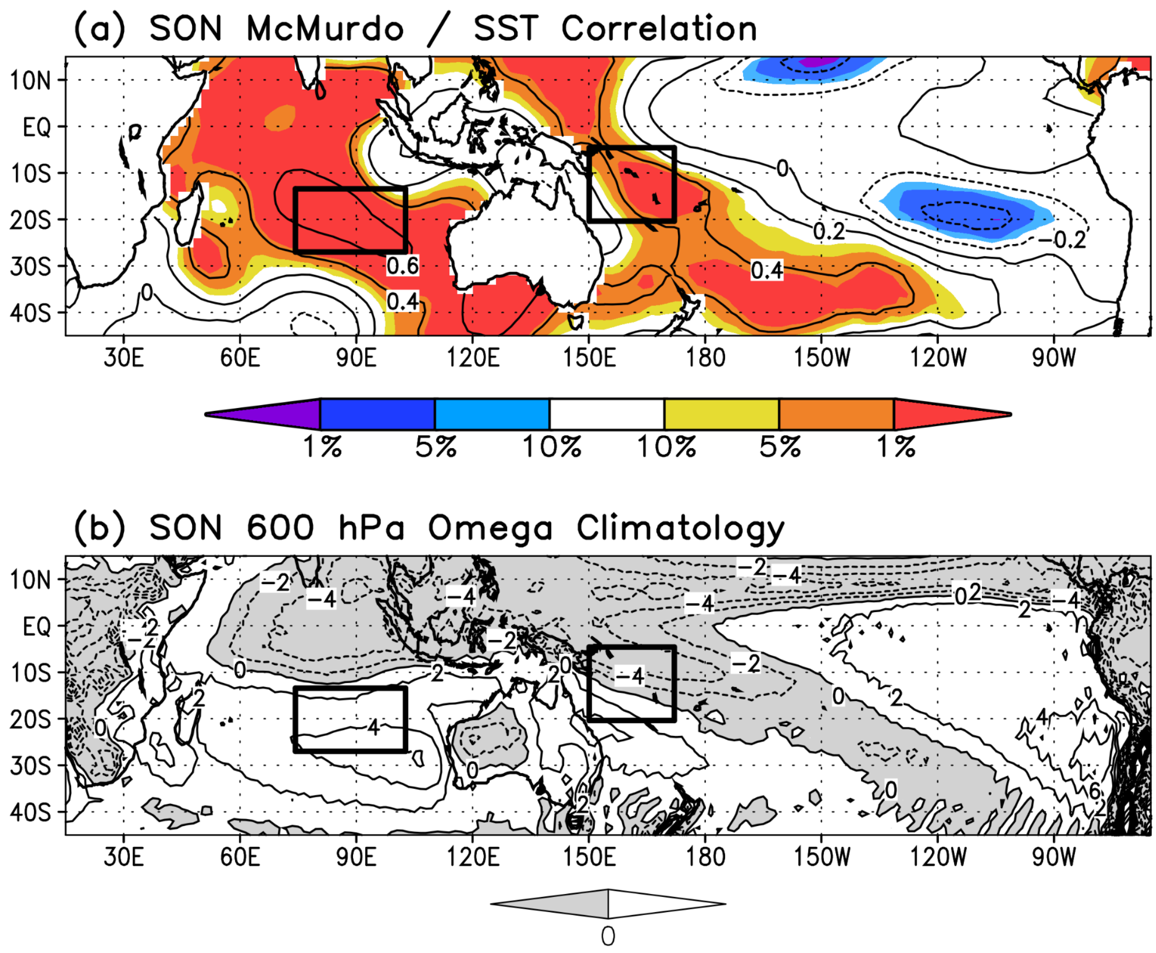

Supplementary Figure 2. Top panel (a) is the SON correlation over 1979-2014 of ERA-Interim McMurdo temperature with ERSSTv4 tropical SST, as in Fig. 4a. Shading in (a) indicates where correlations are significant and is drawn as in Fig. S1. Contour interval in (a) is 0.2. Black boxes denote regions of strong positive correlations statistically significant at $p<0.01$. These boxes indicate regions where positive SST trends would be linearly associated with warming at McMurdo. Bottom panel (b) is the SON 1981-2010 mean for 600 hPa vertical velocity (omega). Grey shading in (b) denotes negative values (regions of climatological ascent) and white regions denote positive values (regions of climatological subsidence). Contour interval for (b) is 2 hPa s^{1}.

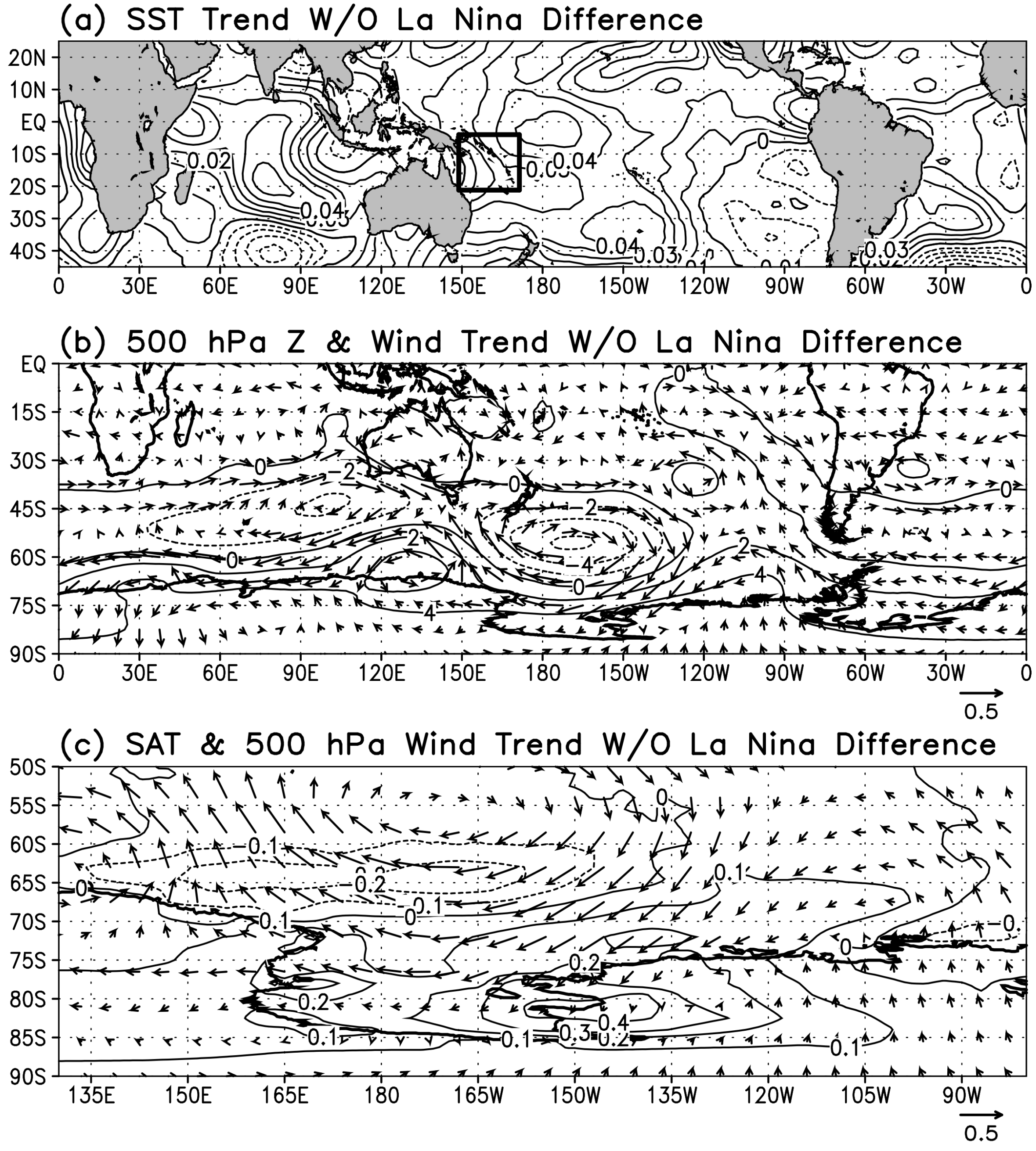

Supplementary Figure 3. SON difference in trends over 1979-2014 after removing La Niña-only years (2010, 1988, 2000, 1998) and retaining negative IPO years thereby showing IPO-related trends without the influence of La Niña-related forcing. Top panel (a) is NOAA's ERSSTv4 tropical SST, (b) Southern Hemisphere ERA-Interim 500 hPa geopotential height and wind, and (c) western West Antarctic ERA-Interim 2 m temperature and 500 hPa wind. Wind vectors are in units of ms^{-1} $decade^{-1}$. Contour interval is 0.01 °C $decade^{-1}$ in (a), 2 m $decade^{-1}$ in (b), and 0.1 °C $decade^{-1}$ in (c).

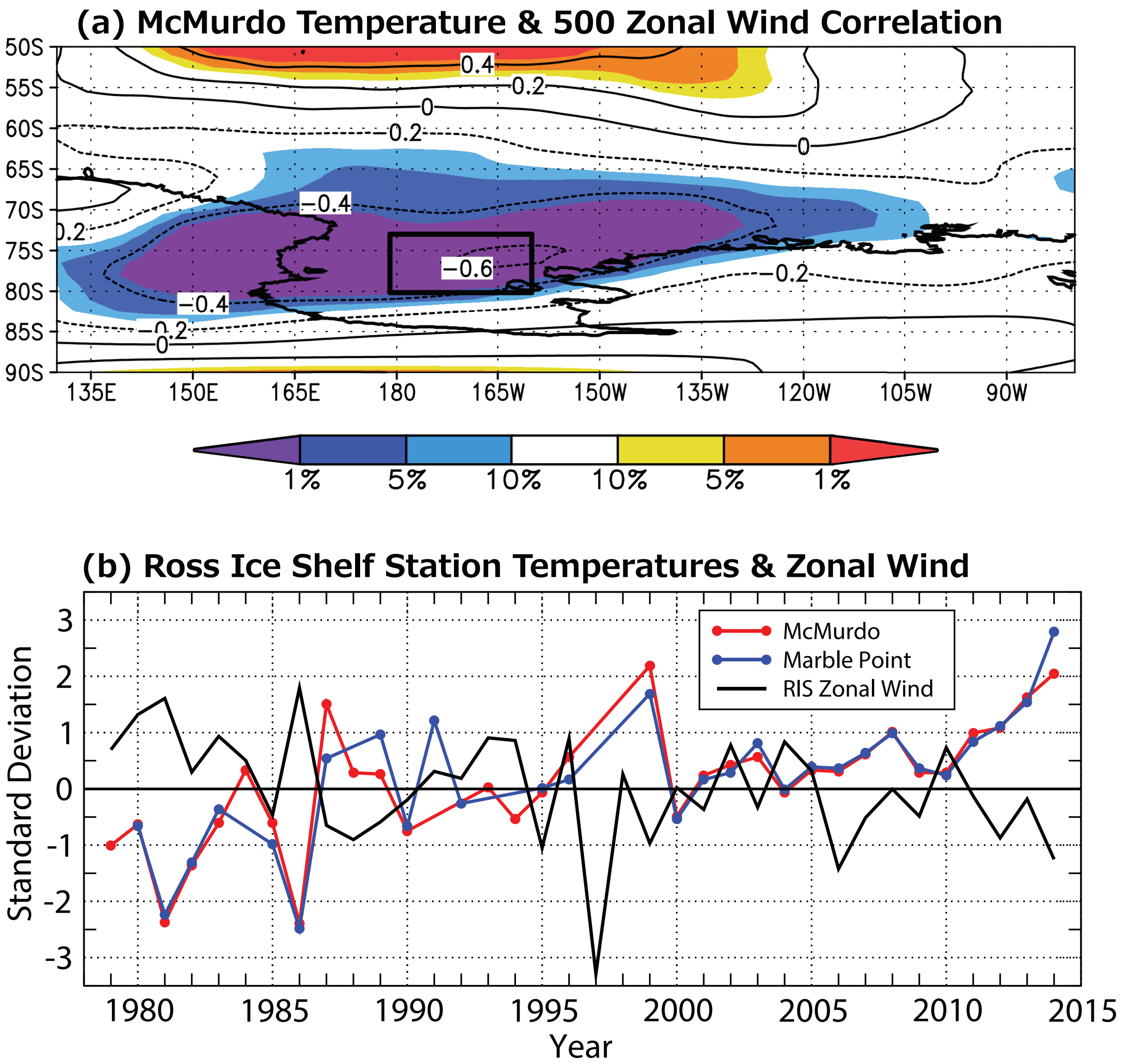

Supplementary Figure 4. SON correlation of western Ross Ice Shelf surface temperature with zonal winds showing Ross Ice Shelf airstream pattern. Top panel (a) is SON correlation over 1979-2014 of ERA-Interim McMurdo temperature with ERA-Interim 500 hPa zonal winds. Bottom panel (b) is the SON standardized time series of McMurdo and Marble Point observed temperatures alongside ERA-Interim 500 hPa zonal wind over the northern Ross Ice Shelf averaged over 180-160.5°W, 73.5-79.5°S (black box in (a)). Shading in (a) indicates statistical significance of correlations and is drawn as in Fig. S2a. Both McMurdo and Marble Point temperatures (red and blue lines in (b)) are correlated with Ross Ice Shelf zonal winds at -0.65 significant at $p<0.01$.
